# Development and Validation of a Scale to Measure Controlling Behaviors in Adolescent Dating Relationships

**DOI:** 10.1177/08862605251355980

**Published:** 2025-07-29

**Authors:** Deziray De Sousa, Alison Paradis, Andréanne Fortin, Mylène Fernet, Martine Hébert, Natacha Godbout

**Affiliations:** 1 Department of Psychology, Université du Québec à Montréal, Canada; 2 Department of Sexology, Université du Québec à Montréal, Canada

**Keywords:** adolescence, controlling behaviors, scale development, validation, teen dating violence

## Abstract

In adolescent dating relationships, controlling behaviors are typically used to alter the partner’s behavior and beliefs. Control has deleterious consequences for the victims’ well-being and has been shown to lead to the perpetration and escalation of other forms of violence (e.g., physical or sexual). Despite the importance of assessing controlling behaviors in adolescent dating relationships, no specific measure is currently available. To address this gap, the current study aimed to develop and validate the *Control in Dating Relationships Scale* (CDRS) which measures controlling behaviors in adolescent dating relationships. Two samples of French-speaking dating adolescents were recruited via social media (*n*_1_ = 311; *n*_2_ = 325). Results of the exploratory factor analyses conducted in the first study revealed a two-factor structure of Isolation (α = .88) and Domination (α = .84), as well as strong temporal stability of scores over 3 months for both Isolation (*r* = .63) and Domination (*r* = .59). These results were cross-validated in the second sample, with evidence of convergent validity and support for measurement invariance across the victimization and perpetration scales. The CDRS is a promising tool to aid researchers in assessing the presence of controlling behaviors in adolescent dating relationships and, consequently, help inform dating violence prevention programs on controlling behaviors, their consequences, and the risk of escalation into other forms of violence.

## Introduction

In adolescent relationships, controlling behaviors have been found to often occur in the absence of other forms of violence (Catallozi et al., 2011; Frye et al., 2006; Gage & Hutchinson, 2006; O’Leary & Smith Slep, 2003) and to have detrimental impacts on the socio-emotional well-being of victims (Glass et al., 2003; Taquette & Monteiro, 2019). This is concerning as authors have observed that, even without the use of physical violence, control can be associated with imminent fear, which often persists even following separation, with victims fearing for their safety (Conroy & Crowley, 2022; Crossman et al., 2016). Furthermore, studies have also revealed that controlling behaviors are a strong predictor of other forms of interpersonal violence (e.g., physical; Fawson, 2015; Graham-Kevan & Archer, 2008). These findings underscore the importance of specifically studying controlling behaviors as distinct from other forms of interpersonal violence in adolescent dating relationships. While often associated, a clear distinction exists between control and psychological violence, thus making it essential to understand and measure these constructs as distinct forms of violence. According to the World Health Organization (2012), psychological violence includes insults, belittling, humiliation, intimidation (e.g., destroying things), threats to harm, and threats to take away children. On the other hand, controlling behaviors are defined as isolating a person from family and friends, monitoring their movements, and restricting access to financial resources, employment, education, or medical care. Furthermore, the UK home office (2013) defined control as “making a person subordinate and/or dependent by isolating them from sources of support, exploiting their resources and capacities for personal gain, depriving them of the means needed for independence, resistance and escape and regulating their everyday lives.” Though these definitions allow for a distinction between the constructs of psychological violence and control, certain aspects of the definitions are not relevant to dating relationships as adolescents are not financially dependent on one another and do not typically have children.

A definition that can help to guide the conceptualization of control in adolescent relationships is presented by Stark (2007) who defines coercion as “the use of force or threats to compel or dispel a particular response” (p. 228), while control refers to “structural forms of deprivation, exploitation, and command that compel obedience indirectly” (p. 229). When coercion and control occur together, he argues, the result is a “condition of unfreedom” (p. 205) that is experienced as entrapment. Many authors have emphasized the need to examine control as its own form of violence in different populations and relational contexts (Burman & Brooks-Hay, 2018; Fitz-Gibbon & Walklate, 2017; Stark & Hester, 2019; Walby & Towers, 2018). One such context that has yet to be examined in depth is adolescent dating relationships.

The reality of control in adolescence may be different and more difficult to measure as there are not as many external factors that they can manipulate (e.g., kids, finances, household chores, job acquisition, and medical care). However, adolescents may use even more subtle forms of control by manipulating their partners’ emotions (e.g., guilt or fear induction) to get the desired outcome. New relationships can bring about various challenges and insecurities (e.g., a partner talking to someone they could potentially be attracted to) and emotions (e.g., jealousy). Also, controlling behaviors are often perceived by adolescents as a normal and inevitable occurrence in relationships given that, on average, one in three adolescents believes that control allows them to demonstrate their commitment, love, and trust for their partner (Catallozi et al., 2011; De Miguel, 2015). Therefore, when adolescents encounter behaviors or attitudes in a partner that they wish to change, they might resort to more subtle tactics, such as control, rather than using overt forms of violence (e.g., physical, psychological, and sexual), which are less socially acceptable and normalized (Overall et al., 2006; Overall et al., 2013).

In light of this, a qualitative study conducted by De Sousa et al. (2023) identified three main subdimensions of control present in adolescent dating relationships, namely Isolation, Domination, and Emotional Manipulation. Isolation tactics aim to distance the victim from their support system to increase their vulnerability and dependence on the perpetrator (e.g., getting upset and demanding a change in behavior when one’s partner interacts with someone of a different sex). Domination instills a sense of doubt regarding the victim’s decisions and identity as the perpetrator imposes their own views without considering those of their partner, with the goal that the victim will succumb to the perpetrator’s demands to avoid upsetting them or causing a conflict (e.g., using threats to influence a partner’s behavior). Emotional manipulation, on the other hand, is used to alter the victim’s behavior, decisions, or beliefs using tactics such as guilt induction, withdrawal of affection, and ignoring a partner (e.g., threatening to hurt oneself or end the relationship). These findings by De Sousa et al. (2023) underscore the various ways in which control can manifest in adolescent relationships.

An in-depth literature review not only reaffirmed these three subdimensions (i.e., Isolation, Domination, and Emotional Manipulation) but also introduced three supplementary subdimensions: Threats/Fear Induction, Jealousy Hypervigilance, and Sexual Derogation (Conroy & Crowley, 2022; Follingstad et al., 2015; Graham-Kevan & Archer, 2009; Hamel et al., 2015; Karnani & Zelman, 2019; Murphy et al., 2012). With Threats/Fear Induction, the perpetrator uses their partner’s vulnerabilities to condition them to behave a certain way so that they comply with the perpetrators’ demands (Graham-Kevan & Archer, 2009; Hamel et al., 2015). Jealousy Hypervigilance includes questioning a partner’s loyalty to the relationship and whereabouts, getting angry when a partner hangs out with someone they could potentially be attracted to, or checking a partner’s cell phone without consent (Hamel et al., 2015; Murphy et al., 2012). Lastly, Sexual Derogation includes putting down a partner’s attractiveness or sexual performance with the objective of diminishing their self-esteem and confidence (Conroy & Crowley, 2022; Hamel et al., 2015). Hence, based on the abovementioned literature and qualitative research, it is hypothesized that control tactics in adolescent relationships may be comprised of these six constructs.

Until now, various methodological challenges associated with measuring controlling behaviors in adolescent relationships have limited the possibility of conducting quantitative research studies on this phenomenon. First, adolescent studies often use instruments developed for adults, such as the *Revised Controlling Behaviors Scale* (Graham-Kevan & Archer, 2005), the *Psychological Maltreatment of Women Inventory* (Tolman, 1999), and the *Conflict Tactics Scale* (Straus et al., 1996). However, some control tactics used by adults may be less relevant to youth as they typically do not live with their partners and are not financially dependent on them (e.g., threatening to take the children to keep the partner from leaving, exerting financial control). Contexts specific to adolescents are also missing in the tools developed for adults (e.g., differences related to emotional maturity, peer pressure, power dynamics; Conroy & Crowley, 2022; De Sousa et al., 2023). Second, even though controlling behaviors on their own can be associated with important negative consequences for adolescents’ socio-emotional well-being, they have mostly been examined in combination with other forms of intimate partner violence (i.e., physical violence; Messinger et al., 2014; Zweig et al., 2014). This impedes the research on control, thus hindering our ability to assess its prevalence, correlates and to generate research that might help prevent its occurrence.

Recent studies have investigated controlling behaviors within adolescent dating relationships (Cascardi et al., 2023; Persram et al., 2021; Taylor et al., 2021); however, the measures employed fell short of comprehensively assessing the multi-faceted nature of control, either by evaluating a limited range of dimensions or by utilizing a restricted number of items. Consequently, items often focus on the overarching concept of control, lacking the specificity needed to delve into its distinct facets. More specifically, none of these measures examine all six of the control tactics identified in the literature (i.e., Isolation, Domination, and Emotional Manipulation, and Threats/Fear Induction, Jealousy Hypervigilance, and Sexual Derogation). Moreover, tools to measure control are often adapted based on adult measures or even other concepts such as power dynamics (Taylor et al., 2021). Furthermore, studies often merge items pertaining to control and coercion into subscales measuring other forms of violence, therefore, often conflating control with other forms of violence (i.e., psychological and cyber dating violence) and limiting the ability to measure the nuances and key behaviors of control and its various subdimensions (Cascardi et al., 2023; Persram et al., 2021). Therefore, the pressing need persists for a reliable, valid, and concise instrument tailored to dissect the various subdimensions of controlling behaviors unique to adolescent dating relationships. Such a scale would be indispensable in accurately gauging its prevalence, understanding its correlates, and pinpointing risk factors for perpetration.

## Objectives

Given the paucity of research on controlling behaviors in adolescent dating relationships and the importance of addressing control to prevent teen dating violence, the present study sought to better understand whether and how more unique and subtle forms of control are manifested in adolescent relationships. To do so, the current set of studies aimed to develop the *Control in Dating Relationships Scale* (CDRS) and explore its psychometric properties. Study 1 aimed to explore the factor structure of the CDRS and assess the test–retest reliability of the scale. It was hypothesized that six different factors would emerge, namely Threats/Fear Induction, Domination, Jealousy Hypervigilance, Isolation, Sexual Derogation, and Emotional Manipulation. Study 2 was conducted to confirm the factor structure, examine the convergent validity of the instrument, and to examine measurement invariance across the victimization and perpetration scales. Due to the relationship between control and other forms of violence, it is hypothesized that control will be positively associated with all subtypes of teen dating violence (i.e., sexual, physical, relational, emotional, threats; Fawson, 2015; Graham-Kevan & Archer, 2008). Since relationship quality has been shown to be negatively related to other forms of teen dating violence (Fortin et al., 2022; Todorov et al., 2021), it is hypothesized that this construct will also be negatively correlated to control in adolescent dating relationships.

## Study 1

### Method

#### Participants and Procedures

Adolescents between 14 and 18 years old (*M* = 15.97, *SD* = 1.22) were solicited online through social media platforms (i.e., Facebook, Instagram) to participate in a survey on adolescent dating relationships. The sample included 311 French-speaking adolescents who reported currently being in a relationship or having experienced one in the past 12 months. Among the 311 adolescents, 178 identified as cisgender girls (57.2%), 113 as cisgender boys (36.3%), 19 as transgender or gender diverse (i.e., non-binary or questioning; 6.1%), and one chose not to respond (0.3%). Within the sample, 211 participants reported being different-sex oriented (67.8%), 62 indicated that they were pansexual or bisexual (19.9%), 17 identified as same-sex oriented (5.5%), and the remainder were questioning their sexual orientation (4.3%). The majority of the participants considered themselves as Quebecers (78.1%). Among participants, 62.4% were currently in a relationship, and 37.6% were involved in a dating relationship in the past 12 months. Of these relationships, the majority (88.1%) were exclusive (i.e., only having one partner). Participants were invited to respond to questions based on their current or most recent relationship.

Recruitment of participants was voluntary and conducted in the Winter of 2022. Participants were invited to complete a 15-min questionnaire. If eligible, adolescents were entered for a chance to win a 300$ gift card to a shopping mall of their choice. Ethical approval for the project was granted by the institutional research ethics board of the Université du Québec à Montréal (5127_e_2021).

#### Development of the CDRS

Following a process suggested by Bearss et al. (2016), an in-depth literature review (Conroy & Crowley, 2022; Follingstad et al., 2015; Graham-Kevan & Archer, 2009; Hamel et al., 2015; Karnani & Zelman, 2019; Murphy et al., 2012) and an examination of the emerging themes from semi-structured interviews with 39 adolescents (De Sousa et al., 2023) were used to inspire the development of the scale. From this process, six theoretical subconstructs emerged, including Isolation, Domination, Emotional Manipulation, Threats/Fear Induction, Jealousy Hypervigilance, and Sexual Derogation. This process yielded the creation of an initial pool of 36 items measuring control victimization. As mentioned in previous research, social desirability bias is inherent to self-report measures of perpetration (Bell & Naugle, 2007; Swan et al., 2012; Vigil-Colet, 2012). This bias can lead respondents to overreport socially desirable characteristics and underreport socially undesirable ones, thus adjusting their responses based on social norms and potentially affecting the reliability of the data on perpetration. In fact, a study examining women’s victimization and perpetration found the victimization model to be more reliable than the perpetration model (Swan et al., 2012). Therefore, Study 1 focused solely on validating the victimization items of the current scale. Subsequent to this phase, a rigorous content validation exercise ensued, featuring the participation of four esteemed research professionals specializing in the domain of teen dating violence. Their assessment encompassed the evaluation of item relevance and correspondence with the stipulated definitions, which were derived from published literature. Following the validation process, refinements were made to the linguistic formulation of the items, strategically tailored to ensure the comprehensibility of the items for adolescents. Additionally, a strategic restructuring ensued, comprising the addition, deletion, or reallocation of specific items to specific subdimensions. Ultimately, an initial pool of proposed items was developed based on six constructs identified in the literature, which are recognized as important for measuring control in adolescent dating relationships. This initial pool consisted of 36 items divided into six subdimensions: Threats/Fear Induction (9 items), Domination (4 items), Jealousy Hypervigilance (4 items), Isolation (8 items), Sexual Derogation (5 items), and Emotional Manipulation (6 items). Participants had to respond on a four-point Likert scale ranging from 1 (never) to 4 (often—6 times or more) regarding the frequency of specific controlling behaviors experienced in the past 12 months.

## Results

### Exploratory Factor Analysis

To examine the latent structure of the questionnaire, an exploratory factor analysis (EFA) was conducted using the R software (R Core Team, 2021). The descriptive statistics revealed that less than 1.5% of the data was missing on each variable. Missing data were thus managed using full information maximum likelihood (FIML; Arbuckle, 1996; Enders & Bandalos, 2001). Upon initial examination of descriptive statistics, items were removed if they met at least two of the following criteria: mean below 1.20, variance less than 0.40, Kaiser-Meyer-Olkin (KMO), and/or item-total correlations below .50. This process ensured that the retained items exhibited substantial variability, correlations with other items, and significant contributions to the underlying constructs. During this initial examination of criteria, a total of 13 out of the 36 initial items were removed, including all five items from the Sexual Derogation subscale. Thus, instead of a six-factor structure, the EFA was conducted only with the remaining items from the other five subscales (i.e., Threats/Fear Induction, Domination, Jealousy Hypervigilance, Isolation, and Emotional Manipulation).

An initial EFA was conducted with the 23 remaining items. Oblique rotation was implemented to facilitate correlation between the extracted factors, while the minimum residual extraction method was employed to effectively reduce the unexplained variance between the observed variables. Based on a method agreement procedure that compares results from multiple convergence indicators (i.e., optimal coordinates, parallel analysis, Kaiser criterion, and Velicer’s Map), the first 23-item EFA converged toward a 3-factor model. All five items from the third factor had loadings less than 0.40 and cross-loadings greater than 0.30; thus they were removed (Stevens, 2002; Watkins, 2018). Items from the Jealousy Hypervigilance and Isolation subscales converged into one factor, whereas items from the Threat/Fear Induction and Domination subscales constituted the second factor. Items from the Emotional Manipulation subscales split into these two factors. A second EFA was then conducted on the remaining 18 items and resulted in a 2-factor model (see Table 1). Based on their consistent presence in control questionnaires and the specific items included, the two subscales were named Isolation and Domination. To create a shorter scale while adhering to the baseline criterion of excluding items with loadings below 0.40, the six items with the highest loadings from each subscale were selected for the final version. The final model contained 12 total items (6 in each subscale) and explained 48.4% of the variance. The KMO index of 0.91 demonstrates excellent sampling adequacy (range 0.85–0.94 for individual items), and the Bartlett sphericity test was significant indicating that there is a substantial correlation in the data (*c*^2^ = 1,847.87, *p* < .000). The internal consistency of the CDRS subscales was excellent, with omega coefficients of 0.84 (Domination) and 0.88 (Isolation). Overall, 53.6% of participants reported being subject to at least one Isolation tactic by a partner in the past 12 months while a larger percentage, specifically 71.1%, acknowledged experiencing at least one Domination tactic. The most common item of the Isolation subscale included making a partner feel bad for not spending enough time with them (*M* = 1.54, *SD* = 0.87). For the Domination subscale, not upsetting a partner due to fear of their short temper was the most commonly reported tactic by participants (*M* = 1.74, *SD* = 1.01).

**Table 1.** Mean, Standard Deviations, and Factor Loadings of the EFA (*n*^1^ = 311).

Items	*M* (*SD*)	18-Item		12-Item	
		F1	F2	F1	F2
18. [. . .] made me feel guilty for spending time with my friends or family instead of with him.her.	1.42 (0.75)	0.878		0.861	
20. [. . .] got mad at me for hanging out with someone he.she didn’t like or of whom he.she was jealous.	1.50 (0.85)	0.809		0.776	
16. [. . .] got angry when I went out with my friends without him.her (e.g., to a party, dinner).	1.34 (0.76)	0.793		0.796	
10. [. . .] kept me from establishing relationships with people around me.	1.25 (0.60)	0.730		0.661	
12. [. . .] told me that I didn’t spend enough time with him.her to make me feel bad.	1.54 (0.87)	0.690		0.641	
33. [. . .] decided who I could or could not hang out with.	1.21 (0.61)	0.689		0.630	
23. [. . .] told me that I didn’t care about him.her to make me feel bad.	1.37 (0.76)	0.641		—	—
4. [. . .] made me drift apart from my friends.	1.44 (0.79)	0.603		—	—
2. [. . .] expected me to ask him.her for permission before I made certain personal decisions (e.g., about my clothing, purchases, activities).	1.45 (0.78)	0.595		—	—
8. [. . .] made decisions about my daily life without considering what I wanted (e.g., whose house to go to after school).	1.33 (0.69)	0.561		—	—
6. [. . .] told me that if I loved him.her, I wouldn’t do anything to hurt him.her.	1.58 (0.94)	0.536		—	—
35. [. . .] got angry when I talked to someone about our relationship (positive or negative aspects).	1.40 (0.71)	0.483		—	—
3. Without any proof, [. . .] accused me of being unfaithful.	1.26 (0.65)	—	—	—	—
29. I feared that if I upset [. . .], he.she would break up with me.	1.63 (0.97)		0.871		0.879
28. [. . .] made me feel guilty by blaming me for all our relationship problems.	1.47 (0.89)		0.618		0.661
24. I was careful not to upset [. . .] because I feared his.her short temper.	1.74 (1.01)		0.541		0.587
13. To get what he.she wanted, [. . .] threatened to break up with me.	1.21 (0.62)		0.530		0.544
31. [. . .] ignored me so that I would feel guilty and change my behavior.	1.56 (0.88)		0.524		0.557
25. When we had a disagreement, [. . .] imposed his.her view of things on me.	1.73 (1.01)		0.464		0.496
9. [. . .] interrogated me or other people to find out where I was and who I was with.	1.60 (0.89)	—	—	—	—
17. [. . .] criticized my physical appearance.	1.25 (0.64)	—	—	—	—
26. [. . .] talked badly about my family.	1.34 (0.72)	—	—	—	—
32. I feared that if I upset [. . .], he.she would hurt him.herself.	1.35 (0.83)	—	—	—	—
34. [. . .] didn’t stop scaring me even after I asked him.her to stop (e.g., driving fast while he.she was angry).	1.27 (0.69)	—	—	—	—
Eigenvalue		5.73	2.33	3.38	2.43
Variance accounted (%)		31.8	13.0	28.2	20.3
Cronbach’s alpha		.92	.84	.88	.84

*Note.* Coefficients greater than .40 are presented. F1 = Isolation; F2 = Domination.

### Test–Retest

Of the 311 participants, 105 completed the questionnaire a second time after a 3-month period. Test–retest reliability of the CDRS was assessed using interclass correlation coefficients. Independent of the level of statistical significance, Cohen and Cohen (1983) suggest correlations below .30 to be low, correlations between .30 and .50 to be moderate, and correlations above .50 to be high. As shown in Table 2, test–retest reliability was high for both Isolation (*r* = .622, *p* = .001), Domination (*r* = .593, *p* = .001), and the total score (*r* = .625, *p* = .001). Even though studies have demonstrated in both adolescent and adult samples that violence measures have less stability of measurement than “less-sensitive” measures (e.g., alcohol use; Fincham et al., 2008; Vega & O’Leary, 2007), the CDRS demonstrated good reliability over a 3-month period.

**Table 2.** Test–Retest Reliability (*n* = 105).

Scale	Test–retest correlation
Isolation	.622**
Domination	.593**
Total score	.625**

* *p* < .05. **p < .01.

## Study 2

### Method

#### Participants and Procedures

A second sample was recruited to cross-validate the factor structure obtained in the first study. Adolescents were recruited online through social media during the Summer of 2022 using the same eligibility criteria and procedures described in Study 1. The sample consisted of 326 French-speaking dating adolescents aged between 14 and 18 years old (*M* = 15.82, *SD* = 1.15). Of these participants, 183 identified as cisgender females (56.1%), 119 as cisgender boys (36.5%), 21 as transgender or gender diverse (6.4%; i.e., non-binary, questioning), and three wished to not respond (0.9%). The majority of participants were Quebecer (76.4%). Participants reported being different-sex oriented (68.7%), omnisexual or bisexual (16.9%), questioning their sexual orientation (10.1%), same-sex oriented (4.0%), or did not wish to answer (0.3%). The majority (67.5%) of participants were currently in a dating relationship, while 32.5% reported having been involved in a dating relationship in the past 12 months.

### Measures

#### Control Victimization and Perpetration

Utilizing the conclusive scale derived from Study 1, consisting of 12 victimization items, an additional set of 12 parallel perpetration items were incorporated for Study 2. Participants responded using a four-point Likert scale ranging from 1 (*never*) to 4 (*often*—6 times or more). Thus, participants reported both on their partner’s and their own use of various controlling behaviors.

#### Relationship Quality

Relationship quality was measured using the 10-item *Relationship Quality Inventory for Adolescents* (RQI-A; Fortin et al., 2022). Adolescents were invited to rate their relationship quality, including two subscales (i.e., connectedness and commitment) on a five-point Likert scale from 1 (*strongly disagree*) to 5 (*strongly agree*). The internal consistency of the connectedness (α = .90) and commitment (α = .87) subscales was excellent in the sample.

#### Dating Violence

A shortened 10-item version of the *Conflict in Adolescent Dating Relationships Inventory* (CADRI-S; Fernández-González et al., 2012; Wolfe et al., 2001) was used. The CADRI-S measures the number of times adolescents used or were subject to various violent behaviors (i.e., sexual, relational, threats, physical, and emotional violence) in the past 12 months on a four-point Likert scale from “never” to “often” (six times or more). A higher score on this scale signified a greater frequency of dating violence within the previous 12 months. The internal consistency of the short version of the CADRI was good (α = .85; Fernández-González et al., 2012).

## Results

### Confirmatory Factor Analysis

**Figure 1.**
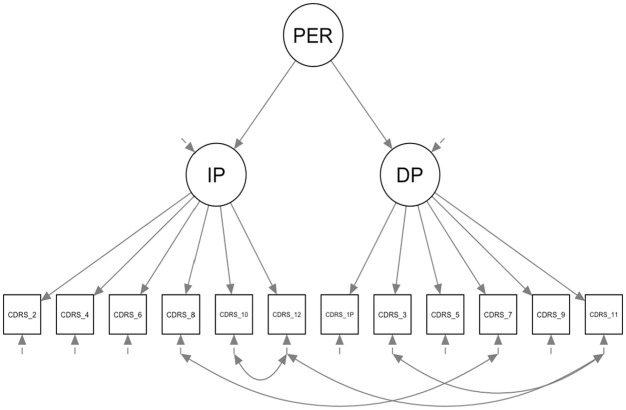
Second-order confirmatory factor analysis for control perpetration. *Note*. PER = control perpetration; DP = domination perpetration subscale; IP = isolation perpetration subscale.

**Figure 2.**
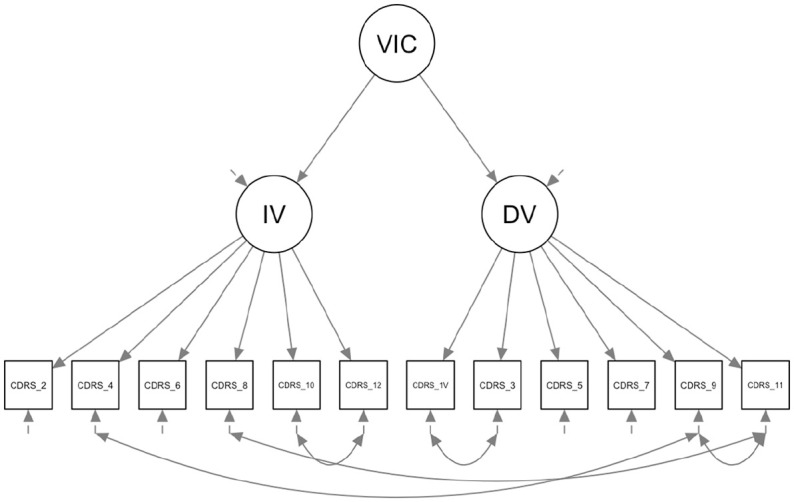
Second-order confirmatory factor analysis for control victimization. *Note*. VIC = control victimization; DV = domination victimization subscale; IV = isolation victimization subscale.

To confirm the factor structure established in the previous study, first- and second-order confirmatory factor analyses (CFA) were conducted for both perpetration (see Figure 1) and victimization (see Figure 2). The second-order CFAs were conducted to explore whether the first-order factors (i.e., Isolation and Domination) could be represented by the overarching second-order constructs (i.e., total score of control victimization and perpetration). Table 3 represents the fit indices of the first- and second-order perpetration and victimization models. The indices examined to assess correspondence between the theoretical and observed models suggested a good fit of the first- and second-order models compared with the expected values: *c*^2^/*df* ratio less than 2, comparative fit index, Tucker-Lewis, and Goodness-of-fit index >0.95, and root mean square error of approximation <0.06 and standardized root mean square residual <0.08 (Hooper et al., 2008; Hu & Bentler, 1999). Moreover, the *R*^2^ values were examined for each subscale, and results indicated that Isolation explained 28.2% of the variance and Domination explained 20.3% of the variance. Within our sample, 31.9% of adolescents reported perpetrating solely controlling behaviors (i.e., without the presence of other forms of direct violence) and 29.1% indicated only being victims of controlling behavior.

**Table 3.** Psychometric Properties of the CDRS (*n*^2^ = 326).

Model	*χ*^2^(*df*)	*χ*^2^/*df*	TLI	RMSEA [90% CI]	SRMR	CFI	GFI (aGFI)
First-order victimization model	61.67 (48)	1.28	0.982	0.036 [0.000–0.061]	.033	0.987	0.981 (0.964)
Second-order victimization model	71.38 (48)	1.48	0.982	0.037 [0.000–0.062]	.033	0.987	0.981 (0.963)
First-order perpetration model	72.87 (49)	1.49	0.946	0.048 [0.022–0.070]	.044	0.960	0.985 (0.973)
Second-order perpetration model	71.38 (48)	1.49	0.945	0.049 [0.022–0.071]	.044	0.960	0.985 (0.972)

*Note*. CDRS = Control in Dating Relationships Scale; CFI = Comparative Fit Index; GFI = goodness-of-fit; RMSEA = root mean square error of approximation; SRMR = standardized root mean square residual; TLI = Tucker-Lewis Index.

### Convergent Validity

Table 4 presents Pearson’s correlations between the CDRS subscales, dating violence subscales, and relationship quality subscales. The victimization and perpetration of both isolation and domination were positively associated with all forms of violence except for the perpetration of Threats. Associations between control and two distinct subscales of relationship quality (i.e., Commitment and Connectedness) as well as a total score were examined. As predicted, the total scale and the subscales of Commitment and Connectedness were negatively associated with Isolation and Domination victimization. Isolation perpetration was negatively associated with Connectedness, but no association was found with the total score or commitment score. Domination perpetration was negatively associated with the total relationship quality and connectedness score yet was not associated with commitment.

**Table 4.** Convergent Validity of the CDRS.

Dating violence and relationship quality	Control perpetration and victimization			
	Isolation victimization	Isolation perpetration	Domination victimization	Domination perpetration
Dating violence (*n* = 319)				
Emotional abuse perpetration	0.382**	0.490**	0.383**	0.508**
Emotional abuse victimization	0.578**	0.439**	0.610**	0.422**
Relational violence perpetration	0.185**	0.246**	0.200**	0.183**
Relational violence victimization	0.418**	0.160**	0.404**	0.178**
Threat perpetration	0.090	0.055	0.050	0.078
Threat victimization	0.348**	0.144*	0.278**	0.185**
Physical violence perpetration	0.161**	0.341**	0.115*	0.238**
Physical violence perpetration	0.434**	0.286**	0.332**	0.261**
Sexual violence perpetration	0.188**	0.119*	0.185**	0.159**
Sexual violence victimization	0.431**	0.173**	0.361**	0.214**
Relationship quality (*n* = 290)	−0.421**	−0.095	−0.298**	−0.227**
Commitment	−0.312**	−0.035	−0.122**	−0.115
Connectedness	−0.467**	−1.95**	−0.415**	−0.297**

*Note*. Point-biserial correlations were computed for dating violence perpetration and victimization (dichotomous variable). CDRS = Control in Dating Relationships Scale.

* *p* < .05. **p < .01.

### Analysis of Invariance

Measurement invariance between the victimization and perpetration scales was assessed by examining changes in χ² between nested models, alongside the commonly accepted criteria of a change of ≤−.01 in CFI combined with a change of ≤.015 in RMSEA (Putnick & Bornstein, 2016). To assess whether the scale measured the same underlying construct across the victimization and perpetration subscales, tests of configural, metric, and scalar invariance were conducted. Configural invariance was supported (Δχ² = .910, ΔCFI = 0.006, ΔRMSEA = −0.008), indicating that the factor structure and pattern of fixed loadings were consistent across groups. Next, metric invariance was tested to assess whether the strength of item–factor relationships was equivalent. Full metric invariance was not supported (Δχ² = .032, ΔCFI = 0.005, ΔRMSEA = −0.007). However, model fit improved after releasing the constraint on Item 6 (Δχ² = 0.174, ΔCFI = 0.001, ΔRMSEA = −0.003), supporting partial metric invariance. Finally, scalar invariance was fully achieved (Δχ² = .067, ΔCFI = 0.003, ΔRMSEA = 0.001), suggesting that observed mean differences in the latent construct reflect true differences in the shared variance of the items.

## Discussion

The objective of the current study was to analyze the validity and reliability of the newly developed CDRS used to measure control in adolescent dating relationships. Drawing from a comprehensive literature review, in-depth qualitative interviews, and a rigorous content validation exercise by four esteemed research professionals, a set of 36 items was created to encompass six distinct constructs of control: Threats/Fear Induction, Domination, Jealousy Hypervigilance, Isolation, Sexual Derogation, and Emotional Manipulation. The underlying assumption was that these constructs would materialize as six separate factors in the current analysis. However, the ensuing results underscored a noteworthy revelation. Out of the original six dimensions, only 12 items from two principal constructs, namely Isolation and Domination, emerged as significant components of controlling behaviors in adolescent dating relationship.

Precisely, all items from the Sexual Derogation subscale were removed as a result of the initial selection of items based on descriptive statistics. While the concept of Sexual Derogation, encompassing actions such as ridiculing a partner’s sexual performance, holds significance within the context of control dynamics, it is probable that these items are somewhat delimited to the realm of sexual coercion and diverge from the broader spectrum of items of the current scale. This nuanced differentiation potentially explains the reasoning behind the lack of alignment of these specific items with the overarching construct of control.

Contrary to expectations, an examination of the five remaining theoretical subscales (i.e., Threats/Fear Induction, Domination, Isolation, Jealousy Hypervigilance, and Emotional Manipulation) did not support a five-factor structure; instead, items were reorganized into two broader subscales of Isolation and Domination. Our results found that Jealousy Hypervigilance converged with items of Isolation despite being presented as a separate construct in previous research. Feelings of jealousy are common in adolescent relationships (Adams & Williams, 2014); however, they become problematic when they involve feelings of possessiveness, ownership, and control that interfere with a partner’s daily activities and other relationships (Ferriani et al., 2019). Consequently, when feelings of jealousy arise, this may lead perpetrators to use Isolation tactics (e.g., limit their partner’s contact with others) in order to reduce the distressing emotions experienced. This may explain why the current results revealed a strong interconnection between the constructs of Jealousy Hypervigilance and Isolation. It is possible that adolescents employ Isolation tactics as a strategy to control their partner’s social interactions in order to diminish potential situations that could trigger feelings of jealousy. Similarly, the items from the Threats/Fear Induction and Domination scales converged. Perpetrators using Domination tactics are attempting to alter their partner’s behaviors or beliefs. These demands often become threats as they are attached to stipulations (e.g., ending the relationship; Dutton & Goodman, 2005). Consequently, these results confirm how threats and fear induction are a common tactic used to dominate one’s partner. Finally, the items related to Emotional Manipulation were found to be separated into the Isolation and Domination subscales. Thus, instead of being a tactic of its own, Emotional Manipulation may be used as a way of both isolating and dominating one’s partner.

Results of the current study confirm previous findings that both CDRS subscales (i.e., Isolation and Domination) were positively associated with other forms of dating violence (i.e., emotional, relational, physical, and sexual) perpetration and victimization (Fawson, 2015; Graham-Kevan & Archer, 2008). Interestingly, control victimization was associated with dating violence perpetration. Thus, as mentioned in previous studies, being the subject of control tactics may prompt adolescents to resort to other forms of violence as a form of resistance. As one partner uses controlling behaviors, the other partner may become more aggressive as a way of resisting (Murphyet al., 2012). The Threat perpetration subscale of the CADRI was not significantly associated with Isolation and Domination perpetration and victimization. Moderate correlations were found between control and psychological violence, confirming that these constructs are related but distinct.

Both Isolation and Domination victimization were found to be associated with lower overall relationship quality as well as the specific Connectedness and Commitment subscales in adolescence. This is consistent with the findings of various studies that have demonstrated the association between dating violence and poor relationship quality (Lancaster et al., 2019; Madlock & Westerman, 2011; Orpinas et al., 2013), thus demonstrating the validity of the current measure. As one of the most important predictors of satisfaction in future relationships, relationship quality in dating relationships is essential to adolescents’ well-being (Madsen & Collins, 2011). Interestingly, the perpetration of Isolation tactics was not associated with overall relationship quality or Commitment to one’s relationship. Likewise, domination perpetration was not associated with adolescents’ level of Commitment. It could be hypothesized that adolescents who isolate or dominate their partner are committed to their partner thus, use these maladaptive tactics to ensure the relationship continues. Other studies have found that commitment and dating violence are not always correlated. Individuals who place a high value on their relationship often continue to want to maintain it, even when they encounter difficulties (Fortin et al., 2022; Furman, 2018). The results of the current study extend previous findings on other forms of violence by demonstrating that the presence of controlling behaviors is also associated with the quality of adolescent dating relationships. Furthermore, results of the measurement invariance analysis provide evidence that the factor structure of the CDRS is largely consistent across the victimization and perpetration subscales, supporting its utility in assessing controlling behaviors from both perspectives.

Overall, the current study provided preliminary evidence that the CDRS appears to be a reliable and valid measure of control perpetration and victimization in adolescent dating relationships. However, some limitations must be addressed. First, the sample used to validate the current questionnaire only included French-speaking adolescents recruited from Quebec thus lacking representativity. Also, the current sample included mostly cisgender different-sex oriented adolescents, which limits the ability to generalize the scale’s reliability to adolescents in the 2SLGBTQIA+ community. Consequently, future studies should examine the reliability of the scale in different populations and cultures. Moreover, future studies should examine measurement invariance across diverse groups (e.g., sex, gender identity, and geographical location) in order to further evaluate the scale’s precision in measuring control dynamics. Second, answers to the questionnaire were self-reported, which may have led to a variety of biases related to memory and desirability. Moreover, due to the cross-sectional design of the current study, definitive results regarding cause and effect between control, relationship quality, and other forms of violence cannot be obtained. Thus, future longitudinal studies should incorporate the current scale to determine how various risk factors impact the perpetration of control on a daily basis. Longitudinal studies should also examine the specific context in which control occurs (e.g., joking, controlling, humiliating) to gain a better understanding of the motives as well as the impact control may have on adolescents. Finally, in order to prevent the occurrence of control in adolescent dating relationships, it is important to further examine various aspects, including risk factors for the development of control, consequences for the victim, and specific factors that cause controlling behaviors to escalate into other forms of violence (i.e., physical, sexual, and emotional violence). Future studies should implement this scale into dyadic studies in order to further examine risk factors for the perpetration of control and determine how these dynamics emerge and are maintained.

The CDRS is a promising scale that can be used with adolescents to measure control in dating relationships without the presence of other forms of violence. The need to delve deeper into the study of control is essential due to the various consequences it has on adolescents, who are in a pivotal developmental phase. Furthermore, first relationships are often experienced during this period and set the foundation for all future relationships, making it important to address these maladaptive behaviors early. As discussed in the results section, controlling behaviors are reported by adolescents even in the absence of other forms of violence. This highlights the significance of control not merely as a risk factor for other forms of violence (Conroy & Crowley 2022; Fawson, 2015; Glass et al., 2003; Graham-Kevan & Archer, 2008; Taquette & Monteiro, 2019), but as a distinct issue. Consequently, control is an important construct to study in adolescent relationships to prevent its occurrence and escalation into other forms of violence. By implementing the CDRS into additional studies on dating relationships, research can inform adolescent dating violence prevention programs by incorporating additional information on controlling behaviors. Unlike in other countries such as England and Scotland, controlling or coercive behaviors in abusive relationships are not currently recognized in the Canadian Criminal Code (Stark & Hester, 2019). Consequently, many cases of such control do not get classified in Canada because there is no core charge of assault or a related offense. The current scale can help address this social issue by demonstrating through research the prevalence of these behaviors and their impact on adolescent well-being, as well as encouraging further investigation into controlling behavior in adolescent dating relationships.

## Supplemental Material

sj-docx-1-jiv-10.1177_08862605251355980 – Supplemental material for Development and Validation of a Scale to Measure Controlling Behaviors in Adolescent Dating Relationships Supplemental material, sj-docx-1-jiv-10.1177_08862605251355980 for Development and Validation of a Scale to Measure Controlling Behaviors in Adolescent Dating Relationships by Deziray De Sousa, Alison Paradis, Andréanne Fortin, Mylène Fernet, Martine Hébert and Natacha Godbout in Journal of Interpersonal Violence
