# Supplementary material for: Development and Validation of a Scale to Measure Controlling Behaviors in Adolescent Dating Relationships
Source: J Interpers Violence. 2025 Jul 29;41(15-16):5479–503. doi: 10.1177/08862605251355980 (PMC13373285; doi:10.1177/08862605251355980)
Supplement: sj-docx-1-jiv-10.1177_08862605251355980 – Supplemental material for Development and Validation of a Scale to Measure Controlling Behaviors in Adolescent Dating Relationships [file sj-docx-1-jiv-10.1177_08862605251355980.docx]

**Table 1.**

*Factor Loadings of the EFA (n^1^ = 311)*

| Items | Loadings | | | | | | |
| --- | --- | --- | --- | --- | --- | --- | --- |
|  | 23-item | | | 18-item | | 12-item | |
|  | F1 | F2 | F3 | F1 | F2 | F1 | F2 |
| 18. […] made me feel guilty for spending time with my friends or family instead of with him.her.^E^ | .921 |  |  | .878 |  | .861 |  |
| 20. […] got mad at me for hanging out with someone he.she didn’t like or of whom he.she was jealous.^J^ | .682 |  |  | .809 |  | .776 |  |
| 16. […] got angry when I went out with my friends without him.her (e.g., to a party, dinner).^I^ | .729 |  |  | .793 |  | .796 |  |
| 10. […] kept me from establishing relationships with people around me.^I^ | .784 |  |  | .730 |  | .661 |  |
| 12. […] told me that I didn’t spend enough time with him.her to make me feel bad.^E^ | .667 |  |  | .690 |  | .641 |  |
| 33. […] decided who I could or could not hang out with.^I^ | .571 |  |  | .689 |  | .630 |  |
| 23. […] told me that I didn’t care about him.her to make me feel bad. ^E^ | .573 |  |  | .641 |  | - | - |
| 4. […] made me drift apart from my friends.^I^ | .642 |  |  | .603 |  | - | - |
| 2. […] expected me to ask him.her for permission before I made certain personal decisions (e.g., about my clothing, purchases, activities).^D^ | .434 |  |  | .595 |  | - | - |
| 8. […] made decisions about my daily life without considering what I wanted (e.g., whose house to go to after school).^D^ | .401 |  |  | .561 |  | - | - |
| 6. […] told me that if I loved him.her, I wouldn’t do anything to hurt him.her.^E^ | .533 |  |  | .536 |  | - | - |
| 35. […] got angry when I talked to someone about our relationship (positive or negative aspects).^I^ | .437 |  |  | .483 |  | - | - |
| 3. Without any proof, […] accused me of being unfaithful.^J^ |  |  |  | - | - | - | - |
| 29. I feared that if I upset […], he.she would break up with me.^T^ |  | .871 |  |  | .871 |  | .879 |
| 28. […] made me feel guilty by blaming me for all our relationship problems. ^E^ |  | .648 |  |  | .618 |  | .661 |
| 24. I was careful not to upset [...] because I feared his.her short temper.^T^ |  | .575 |  |  | .541 |  | .587 |
| 13. To get what he.she wanted, […] threatened to break up with me.^T^ |  | .587 |  |  | .530 |  | .544 |
| 31. […] ignored me so that I would feel guilty and change my behavior. ^E^ |  | .563 |  |  | .524 |  | .557 |
| 25. When we had a disagreement, […] imposed his.her view of things on me.^D^ |  | .480 |  |  | .464 |  | .496 |
| 9. […] interrogated me or other people to find out where I was and who I was with.^J^ |  |  | - | - | - | - | - |
| 26. […] talked badly about my family.^I^ |  |  | - | - | - | - | - |
| 32. I feared that if I upset […], he.she would hurt him.herself.^T^ |  |  | - | - | - | - | - |
| 34. […] didn’t stop scaring me even after I asked him.her to stop (e.g., driving fast while he.she was angry).^T^ |  |  | - | - | - | - | - |
| 1. […] threatened to humiliate me if I didn’t do what he.she wanted.^T^ |  |  |  |  |  |  |  |
| 5. […] told me that I was not attractive.^S^ |  |  |  |  |  |  |  |
| 7. […] threatened to hurt me or someone I care about if I didn’t do what he.she wanted.^T^ |  |  |  |  |  |  |  |
| 11. […] made fun of my sexual performance.^S^  14. […] told me that we were meant for each other and that I couldn't live without him.her.^D^ |  |  |  |  |  |  |  |
| 15. When we weren’t together, […] texted or called me to know what I was doing, where I was and who I was with.^J^ |  |  |  |  |  |  |  |
| 17. […] criticized my physical appearance.^S^ |  |  |  |  |  |  |  |
| 19. To get what he.she wanted, […] threatened to hurt him.herself.^T^ |  |  |  |  |  |  |  |
| 21. […] kept me from doing activities I enjoyed (e.g., sports, art, traveling).^I^ |  |  |  |  |  |  |  |
| 22. […] revealed intimate aspects of our relationship to others.^S^ |  |  |  |  |  |  |  |
| 27. […] negatively compared our sex life with his.her previous experiences.^S^ |  |  |  |  |  |  |  |
| 30. […] told me that I should spend less time with my family.^I^ |  |  |  |  |  |  |  |
| 36. […] threatened to reveal personal information about me if I didn’t do what he.she wanted.^T^ |  |  |  |  |  |  |  |
| Eigenvalue | 5.50 | 2.87 | 0.91 | 5.73 | 2.33 | 3.38 | 2.43 |
| Variance accounted (%) | 22.9 | 12.0 | 3.8 | 31.8 | 13.0 | 28.2 | 20.3 |
| Cronbach’s alpha | 0.92 | 0.84 | 0.67 | 0.92 | 0.84 | 0.88 | 0.84 |

*Note.* F1= Isolation, F2= Domination; Coefficients greater than .40 are presented. Superscripts are included at the end of each item to indicate the subscale to which the item originally belonged: T = Threats/Fear Induction, D = Domination, J = Jealousy/Hypervigilance, I = Isolation, S = Sexual Derogation, E = Emotional Manipulation.

**ÉCHELLE DE CONTRÔLE DANS LES RELATIONS AMOUREUSES**

*[CONTROL IN DATING RELATIONSHIPS SCALE]*

**À quelle fréquence toi et [...] avez-vous dit ou fait les choses suivantes au cours des 12 derniers mois ?** [How often have you and [...] said or done the following things in the last 12 months?]

| **Jamais [Never]** | **Rarement**  **(1-2 fois) [Rarely [1-2 times)]** | **Quelques fois**  **(3-5 fois) [Sometimes (3-5 times]** | **Souvent**  **(6 fois ou plus) [Often ( 6 times or more)** |
| --- | --- | --- | --- |
| **1** | **2** | **3** | **4** |

|  | **1** | **2** | **3** | **4** |
| --- | --- | --- | --- | --- |
| 1. J’ai fait attention de ne pas contrarier […], car je craignais son mauvais caractère. [I was careful not to upset [...] because I feared his.her short temper.] | ○ | ○ | ○ | ○ |
| […] a fait attention de ne pas me contrarier, car il.elle craignait mon mauvais caractère. [[…] was careful not to upset me because he.she feared my short temper.] | ○ | ○ | ○ | ○ |
| 2. […] m’a empêché d’établir des relations avec les gens qui m’entourent. [[…] kept me from establishing relationships with people around me.] | ○ | ○ | ○ | ○ |
| J’ai empêché […] d’établir des relations avec les gens qui l’entourent. [I kept […] from establishing relationships with people around him.her.] | ○ | ○ | ○ | ○ |
| 3. Quand nous avons eu un désaccord, […] m’a imposé son point de vue. [When we had a disagreement, […] imposed his.her view of things on me.] | ○ | ○ | ○ | ○ |
| Quand nous avons eu un désaccord, j’ai imposé mon point de vue à […]. [When there was a disagreement, I imposed my view of things onto […].] | ○ | ○ | ○ | ○ |
| 4. […] s’est fâché.e quand je suis sorti.e avec mes ami.e.s sans lui.elle (p. ex. à un party ou un souper). [[…] got angry when I went out with my friends without him.her (e.g., to a party, dinner).] | ○ | ○ | ○ | ○ |
| Je me suis fâché.e quand […] est sorti.e avec ses ami.e.s sans moi (p. ex. à un party ou un souper). [I got angry when […] went out with his.her friends without me (e.g., to a party, dinner).] | ○ | ○ | ○ | ○ |
| 5. [...] m’a fait sentir coupable en me blâmant pour tous les problèmes dans notre relation. [[…] made me feel guilty by blaming me for all our relationship problems.] | ○ | ○ | ○ | ○ |
| J’ai fait sentir [...] coupable en le.la blâmant pour tous les problèmes dans notre relation. [I made […] feel guilty by blaming him.her for all our relationship problems.] | ○ | ○ | ○ | ○ |
| 6. […] s’est fâché.e contre moi parce que j’ai passé du temps avec une personne qu’il.elle  n’aimait pas ou dont il.elle était jaloux.se. [[…] got mad at me for hanging out with someone he.she didn’t like or of whom he.she was jealous.] | ○ | ○ | ○ | ○ |
| Je me suis fâché.e contre […] parce qu’il.elle a passé du temps avec une personne que je n’aimais pas ou dont j’étais jaloux.se. [I got mad at […] for hanging out with someone I didn’t like or of whom I was jealous.] | ○ | ○ | ○ | ○ |
| 7. […] m’a ignoré pour me faire sentir coupable et que je change mon comportement. [[…] ignored me so that I would feel guilty and change my behavior.] | ○ | ○ | ○ | ○ |
| J’ai ignoré […] pour qu’il.elle se sente coupable et qu’il.elle change son comportement. [I ignored […] so he.she would feel guilty and change her.his behavior.] | ○ | ○ | ○ | ○ |
| 8. […] a décidé qui je pouvais ou ne pouvais pas voir. [[…] decided who I could or could not hang out with.] | ○ | ○ | ○ | ○ |
| J’ai décidé qui […] pouvait ou ne pouvait pas voir. [I decided who […] could or could not hang out with.] | ○ | ○ | ○ | ○ |
| 9. J’ai craint que si je contrariais […], il.elle allait mettre fin à notre relation. [I feared that if I upset […], he.she would break up with me.] | ○ | ○ | ○ | ○ |
| […] a craint que s’il.elle me contrariait, j’allais mettre fin à notre relation. [[…] feared that if he.she upset me, I would break up with him.her.] | ○ | ○ | ○ | ○ |
| 10. [...] m’a fait sentir coupable de passer du temps avec mes ami.e.s ou ma famille plutôt  qu’avec lui.elle. [[…] made me feel guilty for spending time with my friends or family instead of with him.her.] | ○ | ○ | ○ | ○ |
| J’ai fait sentir [...] coupable de passer du temps avec ses ami.e.s ou sa famille plutôt qu’avec moi. [I made […] feel guilty for spending time with his.her friends or family instead of with me.] | ○ | ○ | ○ | ○ |
| 11. Pour avoir ce qu’il.elle voulait, […] a menacé de mettre fin à notre relation. [To get what he.she wanted, […] threatened to break up with me.] | ○ | ○ | ○ | ○ |
| Pour avoir ce que je voulais, j’ai menacé […] de mettre fin à notre relation. [To get what I wanted I threatened [...] to break up with him.her.] | ○ | ○ | ○ | ○ |
| 12. [...] m’a dit que je ne passais pas assez de temps avec lui.elle pour que je me sente mal. [[…] told me that I didn’t spend enough time with him.her to make me feel bad.] | ○ | ○ | ○ | ○ |
| J’ai dit à [...] qu’il.elle ne passait pas assez de temps avec moi pour qu’il.elle se sente mal. [I told […] that he.she didn’t spend enough time with me to make him.her feel bad.] | ○ | ○ | ○ | ○ |
